# Optimal sequence of tests for the mediastinal staging of non-small cell lung cancer

**DOI:** 10.1186/s12911-016-0246-y

**Published:** 2016-01-26

**Authors:** Manuel Luque, Francisco Javier Díez, Carlos Disdier

**Affiliations:** 1 Dept. Artificial Intelligence, UNED, Juan del Rosal, 16, Madrid, 28040 Spain; 2 CIBERES (CIBER of Respiratory Diseases), Pulmonary Department, University Hospital, Ramòn y Cajal, 3, Valladolid, 47005 Spain

**Keywords:** Decision making under uncertainty, Cost-effectiveness analysis in medicine, Probabilistic graphical models, Influence diagrams, Bayesian networks

## Abstract

**Background:**

Non-small cell lung cancer (NSCLC) is the most prevalent type of lung cancer and the most difficult to predict. When there are no distant metastases, the optimal therapy depends mainly on whether there are malignant lymph nodes in the mediastinum. Given the vigorous debate among specialists about which tests should be used, our goal was to determine the optimal sequence of tests for each patient.

**Methods:**

We have built an influence diagram (ID) that represents the possible tests, their costs, and their outcomes. This model is equivalent to a decision tree containing millions of branches. In the first evaluation, we only took into account the clinical outcomes (effectiveness). In the second, we used a willingness-to-pay of € 30,000 per quality adjusted life year (QALY) to convert economic costs into effectiveness. We assigned a second-order probability distribution to each parameter in order to conduct several types of sensitivity analysis.

**Results:**

Two strategies were obtained using two different criteria. When considering only effectiveness, a positive computed tomography (CT) scan must be followed by a transbronchial needle aspiration (TBNA), an endobronchial ultrasound (EBUS), and an endoscopic ultrasound (EUS). When the CT scan is negative, a positron emission tomography (PET), EBUS, and EUS are performed. If the TBNA or the PET is positive, then a mediastinoscopy is performed only if the EBUS and EUS are negative. If the TBNA or the PET is negative, then a mediastinoscopy is performed only if the EBUS and the EUS give contradictory results. When taking into account economic costs, a positive CT scan is followed by a TBNA; an EBUS is done only when the CT scan or the TBNA is negative.

This recommendation of performing a TBNA in certain cases should be discussed by the pneumology community because TBNA is a cheap technique that could avoid an EBUS, an expensive test, for many patients.

**Conclusions:**

We have determined the optimal sequence of tests for the mediastinal staging of NSCLC by considering sensitivity, specificity, and the economic cost of each test. The main novelty of our study is the recommendation of performing TBNA whenever the CT scan is positive. Our model is publicly available so that different experts can populate it with their own parameters and re-examine its conclusions. It is therefore proposed as an evidence-based instrument for reaching a consensus.

## Background

Lung cancer is a very common tumor in the developed world and the leading cause of cancer death. Lung cancer can be classified into two major types: small-cell lung cancer and NSCLC. The former, which accounts for 20 % of cases [1], is usually inoperable and treatable only with chemotherapy or chemoradiotherapy. In contrast, when it is limited to the lung, to certain adjacent structures, and to lymph nodes proximal to the lung, NSCLC can be treated with surgical resection. However, more than 80 % of NSCLC patients cannot be treated with surgery because the disease is out of control due to a metastasis [2]. A disappointing fact is that a high percentage of patients that may benefit from surgery die of lung cancer. A correct assessment at an early stage of the disease—the staging phase—would help to determine which patients may benefit from surgery and, in turn, to avoid dangerous, painful, and unnecessary surgery when metastasis has already occurred.

When there are no distant metastases, mediastinal staging, i.e., determining whether malignant mediastinal lymph nodes are present or absent, is the most important prognostic factor in patients with NSCLC and, consequently, determines the therapeutic strategy. Various techniques are available to study the mediastinum, such as non-invasive imaging techniques (CT scan and PET) and minimally invasive endoscopic techniques (TBNA, EBUS, EUS), involving varying degrees of sensitivity and specificity; more invasive surgical techniques include mediastinoscopy. The main treatment options for lung cancer include surgery, chemotherapy, radiation therapy, chemoradiotherapy, palliative and supportive care, and no treatment. The applicability of each treatment depends on the stage of the tumor. Due to the variety of available tests and treatments for NSCLC, each one with its pros and cons and different economic costs, there is a vigorous debate among specialists about which tests and treatments should be used to strike a balance between *effectiveness* and*costs* [3, 4].

In an attempt to clarify this controversy using an evidence-based approach [5–7], we have built an ID for this problem from the perspective of the Spanish public health system. The ID was evaluated twice, first without considering economic costs, and then by converting costs into effectiveness using a willingness-to-pay of €30,000 per QALY, the shadow threshold estimated for that health system [8, 9]. We performed several types of sensitivity analysis to study the effect of the uncertainty in the numerical parameters of the model.

This paper has been written following the Consolidated Health Economic Evaluation Reporting Standards [10].

## Methods

### Preliminaries

This section describes IDs, the explanation capabilities available in the software tool used to build the model, and the basic principles of cost-effectiveness analysis in medicine.

#### Influence diagrams

Decision trees [11] are a traditional framework for modeling decision problems in medicine. Since decision trees explicitly represent all the possible decision scenarios, the size of the model grows exponentially with the number of variables. That combinatorial explosion makes the use of decision trees prohibitive for medium or large problems.

IDs [12, 13] arose as an alternative to decision trees. Their compactness, based on a causal graph, eases communication with experts, simplifies the solution and debugging, and thus makes IDs appropriate for much larger decision problems.

We start by considering an example of a medical ID. A physician has to decide whether to treat or not a patient, who may suffer from a disease (*X*). Before deciding how to treat the patient (*D*), the physician can perform a test (decision *T*), whose result (*Y*) will help determine whether the patient suffers from the disease. The overall effectiveness results from substracting the morbidity of the test (*U*_1_) from the quality of life that results from treating the patient (*U*_2_).

Formally, an ID consists of an acyclic directed graph having three disjoint sets of nodes: decision nodes **V**_*D*_ (graphically represented by squares or rectangles), chance nodes **V**_*C*_ (circles or ovals), and utility nodes **V**_*U*_ (diamonds or hexagons). Decision nodes represent the actions under the direct control of the decision maker. Chance nodes represent uncertain events. In medical IDs, utility nodes represent medical outcomes and costs (quality of life, morbidity, mortality, economic cost...). Here, two types of utility nodes are distinguished: *ordinary,* having parents that are chance and decision nodes, and *super-value* (SVN), having parents that are other utility nodes [14]. Given that each node represents a variable, we will use the concepts of node and variable interchangeably. We assume that all the chance and decision variables are discrete.

IDs contain three types of arcs, depending on the type of node they go into. Arcs into chance nodes represent probabilistic dependencies. Arcs into decision nodes represent availability of information or precedence relations between decisions. Arcs into ordinary utility nodes indicate the domain of the associated utility function; arcs into a SVN *U* indicate that the associated utility function is a combination of the utility functions of the parents of *U*.

**Fig. 1:**
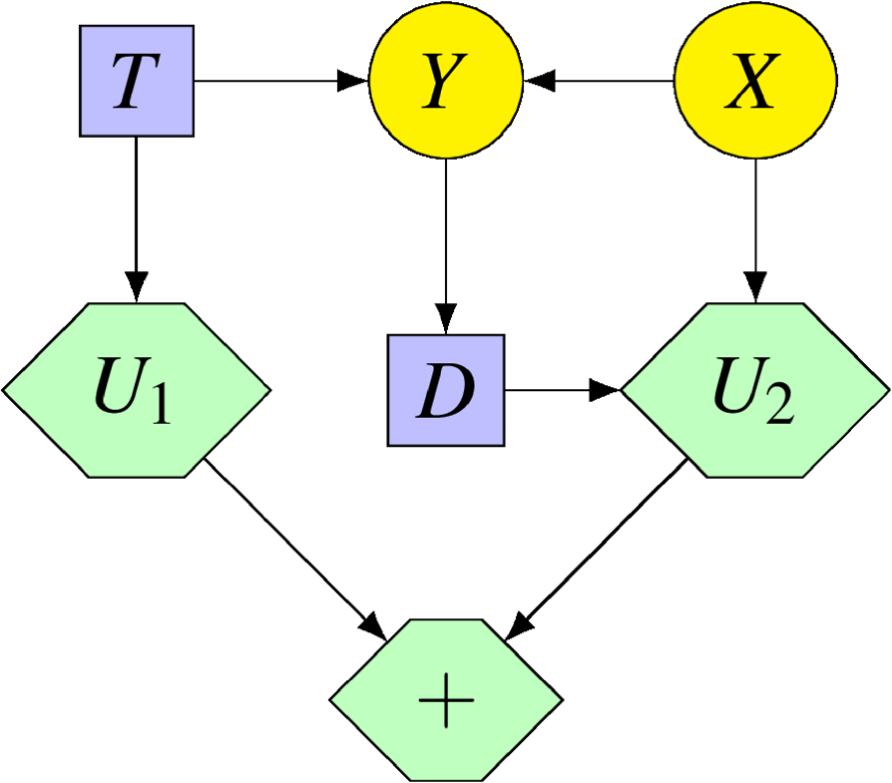
Example of medical influence diagram. A patient may suffer from a disease (*X*). Before deciding how to treat the patient (*D*), the physician can decide to perform a test (*T*). This test will produce the test result (*Y*), which would help to determine whether the patient suffers from the disease. The doctor has to select a strategy taking into consideration the morbidities associated to the test (*U*_1_) and the health state of the patient after treating him (*U*_2_)

For example, in Fig. 1 *X* and *Y* are chance nodes, *T* and *D* are decision nodes, *U*_1_ and *U*_2_ are utility nodes, and the child of *U*_1_ and *U*_2_ is a SVN of type sum. Node *X* has a causal and probabilistic relationship with node *Y*. Variable *X* is not observable, is unknown when making decision *D* and there is thus no arc from node *X* to node *D*. However, variable *Y* is observable, its values are known when making decision *D*, and it can therefore be observed by the decision maker at that moment. This explains the arc pointing *Y* to node *D*.

We assume a path connects all the decision nodes, indicating the order in which decisions are made. Let *n* be the number of decisions in the ID. The total order of decisions {*D*_0_,*D*_1_,…,*D*_*n*−1_} partitions the set of chance variables into the collection of sets {**C**_0_,**C**_1_,…,**C**_*n*_}, where **C**_*i*_, 0≤*i*<*n*, is the subset of chance variables known for *D*_*i*_ but unknown for any previous decision, and **C**_*n*_ is the set of unknown chance variables. Furthermore, the *no-forgetting* hypothesis [15] is assumed, which states that the decision maker recalls all the previous decisions and observations.

For example, in Fig. 1 decision *T* precedes decision *D*, i.e., *D*_0_=*T* and *D*_1_=*D*. This order partitions the set of chance variables in the following way: $\mathbf {C}_{0}=\varnothing, \mathbf {C}_{1}=\{Y\}$ and **C**_2_={*X*}. This partition has a simple interpretation: no chance variable is observed before deciding on *T*, variable *Y* is observed after deciding on *T* but before deciding on *D*, and variable *T* is unknown or observed after deciding on *D*. Thus, when deciding on *D*, the decision maker knows the values of *T* and *Y*.

The quantitative information that defines an ID is given by (1) assigning to each chance node a conditional probability, (2) assigning to each ordinary utility node a real-valued function, and (3) assigning to each SVN a utility-combination function. Conditional probabilities and utility functions of ordinary utility nodes are represented as tables. In the example, the quantitative information consists of the conditional probabilities *P*(*x*) and *P*(*x*|*y*) and the utility functions *ψ*_1_(*x,d*) and *ψ*_2_(*t*).

The *optimal policy* for a decision *D* is a function that maps each configuration of the variables known in *D* onto the option of *D* that maximizes the expected utility. The purpose of evaluating an ID is to compute an *optimal strategy* composed of a set of optimal policies, one for each decision in the ID.

It is well known that inference in probabilistic graphical models, such as Bayesian networks and IDs, is an NP-hard problem [16, 17]. Although the set of decisions is totally ordered in IDs, the search space of an ID solution algorithm grows exponentially with the number of variables. However, there are algorithms and software packages that can evaluate BNs and IDs for many real-world problems in less than a second.

In this paper, the ID was built using Elvira and OpenMarkov, two software tools for probabilistic graphical models [18, 19]. Debugging a probabilistic expert system based on an ID is very difficult because the output of the evaluation of a medium or large problem may be the result of thousands of mathematical operations such as sums, products, divisions, and maximizations. Explaining the reasoning is a key factor in the acceptance of expert systems in real-world domains like medicine. One example consists of explaining why the system recommends one action instead of another. Lacave et al. [20, 21] described how the explanation capabilities of Elvira were useful for building probabilistic medical models and how such capabilities can help make the probabilistic reasoning more understandable to human users. However, it is a very difficult task that has not yet been completely solved in the field of IDs [20].

#### Cost-effectiveness analysis in medicine

If economic costs are included in a medical decision problem, the result is a problem with two criteria to optimize: effectiveness, measured in clinical units, usually QALYs, which we want to maximize, and cost, measured in monetary units, which we want to minimize^1^. In medicine, this problem is typically solved with cost-effectiveness analysis [22].

The goal of cost-effectiveness analysis is to maximize the *net health benefit* (NHB) [23], which is defined as follows:

(1) $$ {NHB}=E-C/\lambda\;,  $$

where *E*is effectiveness, *C* is cost^2^, and *λ* is used to convert effectiveness into cost or vice versa. Parameter *λ* is sometimes called *willingness to pay* and represents the maximum amount of money an individual is willing to sacrifice to gain a unit of effectiveness (health benefit). The value of *λ* is always positive but depends on each decision maker.

### Construction of the model

This section describes the construction of MEDIASTINET, an ID for determining the optimal sequence of tests for the mediastinal staging of NSCLC. The model was developed with the help of a pneumologist during several oral interviews; the pneumologist is the third author of this paper. The perspective adopted in the model was that from the Spanish public health system. The primary decision maker is the Spanish Ministry of Health. Our model applies to patients that have lung cancer, presumably operable, with no distant metastases.

The model assumes that a CT scan is always performed. Silvestri et al. [24] state that a “CT scan is clearly an imperfect means of staging of the mediastinum, but it remains the best overall anatomic technique for studying the thorax”. Additionally, the authors highlight the importance of CT scan, a non-invasive test, for guiding the choice of nodes for the most invasive techniques.

#### Structure of the graph

**Fig. 2:**
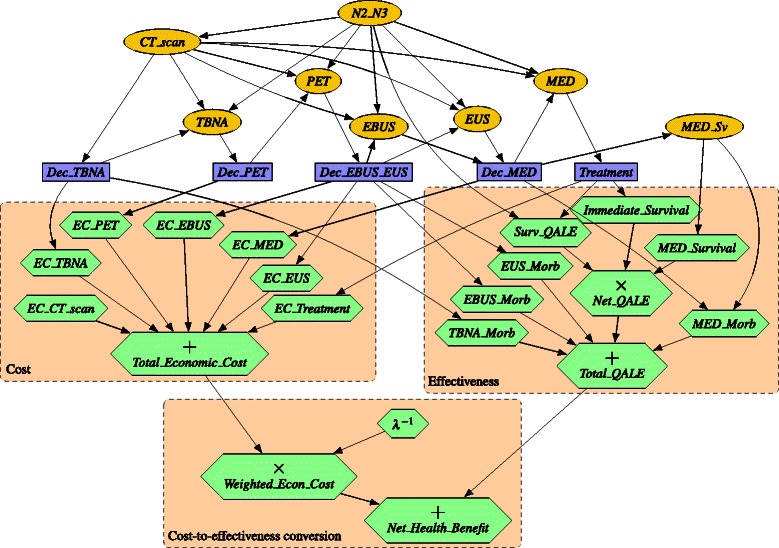
Influence diagram MEDIASTINET. Chance nodes (ovals), except *N2_N3* and *MED_Sv*, correspond to the laboratory tests that can be performed. Decision nodes (rectangles) correspond to the decision on the treatment and on whether to perform each laboratory test. Utility nodes (hexagons or diamonds) have been grouped into three sets, each one surrounded by an orange rectangle background. The first group represents effectiveness, measured in QALYs. The second group of utility nodes represents cost, measured in €. The third group relates cost and effectiveness. Node *λ* ^−1^ represents (the inverse of) the willingness to pay

The graph structure of the ID (see Fig. 2) was built manually following the expert’s knowledge of the variables involved in the problem and the causal relations between them.

##### Chance variables

The TNM classification uses three factors (T, N, and M) to describe the extent of a cancer. The N factor indicates whether regional lymph nodes are affected. Given that the objective is the mediastinal staging of NSCLC, the value of N [1] has been represented by the chance variable *N2_N3*. Even though N takes on four possible values, from N0 to N3, it has been modeled here as a binary variable because cancers are operable for groups N0 and N1, and inoperable for most of N2 and all of N3. The laboratory tests that can be performed are represented by binary variables *CT_scan*, *TBNA*, *PET*, *EBUS*, *EUS*, and *MED* (mediastinoscopy). Binary variable *MED_Sv* represents whether the patient survives mediastinoscopy.

Nodes representing test results (*CT_scan*, *TBNA*, *PET*, *EBUS*, *EUS, EUS*, and *MED*) have a causal and probabilistic relationship with node *N2_N3*. This justifies the arcs pointing from *N2_N3*. Moreover, we have drawn arcs from *CT_scan* to the other test result variables because the *CT_scan* result can influence their sensitivity and specificity.

##### Decision variables

Each decision on whether to perform a laboratory test has been represented by a variable with the prefix *Dec_*on its name. A node *Dec_CT_scan*was not included because a CT scan is always done. The decision maker can perform the EBUS and EUS separately, or both at the same time; this is represented by node *Dec_EBUS_EUS*. These decisions forced the addition of a new state *no_result* to the variables *TBNA*, *PET*, *EBUS*, *EUS*, and *MED*, to reflect the fact that when a test is not performed, its result is not available. Variable *Treatment* represents the set of possible treatments; its states are *thoracotomy*, *chemoradiotherapy*, and *no_treatment.*

We included a node *Treatment* because the effectiveness of the tests is due to the guide they offer on which treatment to apply.

##### Utility nodes

Utility nodes in the ID of Fig. 2 can be grouped into three sets, each surrounded by a dashed rectangle.

The first group represents effectiveness, measured in QALYs. The node *Total_QALE* accumulates the overall *quality adjusted life expectancy*(QALE) of patients [25]. This node sums the morbidities due to medical tests, and the utility function of *Net_QALE*. This last node represents the QALE of patients considering that they can die due to the treatment or to the mediastinoscopy, and is the product of three nodes: *Surv_QALE* represents the QALE of the survivors of the tests and treatments; *MED_Survival* indicates whether the patient survives the mediastinoscopy; and *Immediate_Survival* represents the immediate survival rate after the treatment.

The second group of utility nodes represents cost. *Total_Economic_Cost* is the total cost. Its parents in the graph represent the costs of tests and treatments.

Finally, the third group relates cost and effectiveness: *λ* ^−1^ represents (the inverse of) the willingness to pay, *Weighted_Econ_Cost* represents *λ* ^−1^·*C*, and *Net_Health_Benefit* the NHB (Eq. 1). The reason for using *λ* ^−1^ instead of *λ* is to be able to obtain the optimal policy regardless of cost, as shown below.

Table 1 presents a list of all the variables, along with the type of variable (chance, decision or utility), the domain (discrete or continuous), and the set of possible values (domain).

**Table 1:** Variables of the influence diagram, along with their type, the domain type and the values they can take

Name	Type	Domain type	Domain
*N2_N3*	Chance	Discrete	*positive*; *negative*
*CT_scan*	Chance	Discrete	*positive*; *negative*
*TBNA*	Chance	Discrete	*positive*; *negative*;
			*no_result*
*PET*	Chance	Discrete	*positive*; *negative*;
			*no_result*
*EBUS*	Chance	Discrete	*positive*; *negative*;
			*no_result*
*EUS*	Chance	Discrete	*positive*; *negative*;
			*no_result*
*MED*	Chance	Discrete	*positive*; *negative*;
			*no_result*
*MED_Sv*	Chance	Discrete	*yes*; *no*
*Dec_TBNA*	Decision	Discrete	*yes*; *no*
*Dec_PET*	Decision	Discrete	*yes*; *no*
*Dec_EBUS_EUS*	Decision	Discrete	*ebus* + *eus*;*ebus*;*eus*;
			*no_test*
*Dec_MED*	Decision	Discrete	*yes*; *no*
*Treatment*	Decision	Discrete	*thoracotomy*;
			*chemoradiotherapy*;
			*no_treatment*
*EC_CT_scan*	Utility	Continuous	$\mathbb {R}^{+}$
*EC_TBNA*	Utility	Continuous	$\mathbb {R}^{+}$
*EC_PET*	Utility	Continuous	$\mathbb {R}^{+}$
*EC_EBUS*	Utility	Continuous	$\mathbb {R}^{+}$
*EC_EUS*	Utility	Continuous	$\mathbb {R}^{+}$
*EC_MED*	Utility	Continuous	$\mathbb {R}^{+}$
*EC_Treatment*	Utility	Continuous	$\mathbb {R}^{+}$
*TBNA_Morb*	Utility	Continuous	$\mathbb {R}^{-}$
*EBUS_Morb*	Utility	Continuous	$\mathbb {R}^{-}$
*EUS_Morb*	Utility	Continuous	$\mathbb {R}^{-}$
*MED_Morb*	Utility	Continuous	$\mathbb {R}^{-}$
*Immediate_Survival*	Utility	Continuous	[ 0,1]
*MED_Survival*	Utility	Discrete	0; 1
*Surv_QALE*	Utility	Continuous	$\mathbb {R}^{+}$
*λ* ^−1^	Utility	Continuous	$\mathbb {R}^{+}$

#### Elicitation of probabilities and utilities

There are two types of uncertainty in a model.*First-order uncertainty* reflects that the outcomes of some variables are not under the control of the decision maker. For example, even if a doctor knows that the prevalence of a disease is 0.05, s/he cannot know with certainty whether a person randomly chosen has the disease. *Second-order uncertainty* reflects that the parameters of the probability distribution are not known with certainty [26, 27]. For example, a doctor may not know with certainty the value of the prevalence of a disease, but s/he could assume that its distribution follows a Beta with parameters *α*=5 and *β*=95. In this example, the prevalence of the disease is a parameter of the model, while *α* and *β* are the parameters of the associated second-order distribution.

The next step in the construction of the model was to complete the quantitative part of the ID, consisting of a set of probability and utility potentials. There were 46 independent parameters. Each parameter of the model, except costs, had an associated second-order probability:

- Beta distributions were assigned to the non-extreme probabilities of the prevalence of the disease, the sensitivities and the specificities of tests, and the survival rates, as the data that inform each probability parameter of the model are binomial (*m* cases of interest are observed from a set of *n* observations) [27], and the Beta distribution is the conjugate to binomial data [28].
- Uniform distributions were assigned to the morbidities of the tests. As the literature provided us with the percentage of patients suffering the morbidities and the medical expert said that the effects only last for a month after the test, we subjectively assumed that the quality of life of patients due to the morbidities follow a uniform distribution in the interval [ 0,1]. We have accordingly selected in this case the type of distribution with maximum entropy. This estimation of the quality of life is imprecise, but the subsequent sensitivity analysis demonstrated that the calculated strategy was not sensitive to the uncertainty in the morbidity parameters.
- Although *λ* was known with certainty, we attached it a second-order distribution in order to analyze its variations in the sensitivity analysis phase. A Gamma distribution was assigned to *λ*, as it is constrained in the interval [ 0,+*∞*) [27] and it can be interpreted as a cost. Briggs et al. [27] suggest the use of gamma for costs because the count data of costs is often usually represented by the Poisson distribution, and the Gamma is the conjugate to the Poisson.

The parameters of the second-order distributionswere estimated from the medical literature. Different methods were used to fit these distributions. In the case of Beta distributions, four different methods were used depending on the values provided by the literature:

1. If the source indicated that *m* cases of interest were observed from a set of *n* observations, then we elicited a Beta with shape parameters *α*=*m* and *β*=*n*.
2. If the source only provided mean *μ*, we set a coefficient of variation *k*, and we assigned the standard deviation as *σ*=*k*·*μ*. Then by using the method of moments [27] we calculated the shape parameters *α* and *β*: *z*=(*μ*·(1−*μ*)·*σ* ^2^)−1, *α*=*z*·*μ*, *β*=*z*−*α*. We have taken the value *k*=1/5, following Bond et al. [29, 30] in a study commissioned by the National Institute for Health and Care Excellence (NICE). If *μ* was so close to 1 that it was inconsistent to have a Beta with such parameters *μ* and *σ*, then we took the value *k*=10^−*m*^·1/5 for setting *σ*, where *m* is the minimum natural number guaranteeing consistency.
3. If the source provided mean *μ* and the 95 % confidence interval (CI) [ *l,u*] of the parameter, we found a Beta distribution with mean *μ* so that the interval [ *l,u*] accumulated a 95 % of the probability mass. Given mean *μ* and considering *α* an independent parameter, we have *β*=*α*·(1−*μ*)/*μ*. Then we used a bisection method to find the value of *α* that fulfills *F*(*u*)−*F*(*l*)=0.95, being *F* the cumulative distributive function of Beta.
4. If the source provided mean *μ* and two values *l* and *u*, the latter being the maximum and the minimum values respectively, but the authors did not indicate that *l* and *u* referred to the 95 % CI, then by assuming that the interval [ *l,u*] accumulates 99.9 % of the probability mass we elicited the distribution analogously to point 3. This value is arbitrary but does not significantly affect the sensitivity analysis results.

In the case of the Gamma distribution of *λ*, we followed an approach similar to case 3 above: a mean *μ* was obtained from the source, and we assigned the standard deviation *σ*=*k*·*μ*, being *k* the coefficient of variation explained above.

Tables 2, 3, 4, 5 and 6 present all the independent parameters. Subindices attached to the CIs indicate the type of distribution and the type of elicitation used: numbers 1 to 4 correspond to a point above for the Beta distribution, and number 5 corresponds to uniform distributions.

**Table 2:** Sensitivities and specificities of the tests when CT scan is positive

	Sensitivity		Specificity		
	Mean	CI	Mean	CI	Source
TBNA	78	[40.9, 98.5]_2_	99.5	[94.3, 100]_2_	[24]
PET	91	[86.7, 94.5]_4_	78	[71.9, 83.6]_4_	[56]
EBUS	92.5	[25.5, 100]_2_	99.5	[94.3, 100]_2_	[57]
EUS	90	[32.5, 100]_2_	99.5	[94.3, 100]_2_	[58]
MED	83	[39.9, 99.9]_2_	99.9	[100, 100]_2_	[24]

Mean and 95 % CI values are given in percentages. Subindices denote the elicitation method (see Section “Elicitation of probabilities and utilities”)

**Table 3:** Sensitivities and specificities of the tests when CT scan is negative

	Sensitivity		Specificity		
	Mean	CI	Mean	CI	Source
TBNA	4	[2.6, 5.7]_2_	97.1	[89.7, 99.9]_1_	[59]
PET	75	[66.2, 82.9]_4_	93	[92.4, 93.6]_4_	[56]
EBUS	69.2	[39.3, 91.9]_2_	99.5	[94.3, 100]_2_	[60, 61]
EUS	58	[34.7, 79.5]_2_	99.5	[94.3, 100]_2_	[58]
MED	47	[28.9, 65.5]_2_	99.9	[100, 100]_2_	[24]

Mean and 95 % CI values are given in percentages. In the case of the sensitivity and the specificity of the EBUS, we considered that the mean value of the distribution was the average of the values given by the two references

**Table 4:** Morbidities and costs of tests

	Morbidity			Cost		
	Mean	CI	Source	Mean	CI	Source
CT_scan				199	—	[62, 63]
TBNA	0.000108	[0.000005, 0.000211]_6_	[64]	80	—	[65]
PET				1290	—	[62, 63]
EBUS	0.000021	[0.000001, 0.000041]_6_	[66]	620	—	[67, 68]
EUS	0.000125	[0.000006, 0.000244]_6_	[66]	620	—	[67, 68]
MED	0.000833	[0.000042, 0.001625]_6_	[69]	3000	—	[65]

Moribidities are given in QALYs and costs are given in €

**Table 5:** QALE depending on the treatment, given in QALYs

	pos. N2N3			neg. N2N3		
	Mean	CI	Source	Mean	CI	Source
Thoracotomy	1.17	[0.78, 1.56]_5_	[70]	5.75	[5.36, 6.14]_5_	[71]
Chemoradiotherapy	1.25	[0.86, 1.64]_5_	[70]	2.64	[2.25, 3.03]_5_	[72]
No treatment	0.42	[0.03, 0.81]_5_	[73]	2.08	[1.69, 2.47]_5_	[73]

**Table 6:** Mean and CI of the remaining parameters of the model

	Mean	CI	Source
Prevalence of *N2_N3*	30	[19, 42.4]_2_	[24]
Sensitivity of *CT_scan*	55	[33.2, 75.8]_2_	[24]
Specificity of *CT_scan*	81	[40.5, 99.5]_2_	[24]
*MED* survival rate	99.9	[99.9, 99.9]_2_	[69]
Thoracotomy immediate survival rate, pos. *N2_N3*	96	[93.5, 97.9]_3_	[71]
Thoracotomy immediate survival rate, neg. *N2_N3*	97.8	[92.7, 99.9]_2_	[74]
Chemoradiotherapy immediate survival rate	98	[92.7, 99.9]_2_	[71]
Cost of thoracotomy	9764.4	—	[75]
Cost of chemoradiotherapy	4142.6	—	[75]
*λ*	30000	—	[8, 9]

All parameters, except costs and *λ*, are given in percentages. Costs are given in €. The value of *λ* is given in €/QALY

**Fig. 3:**
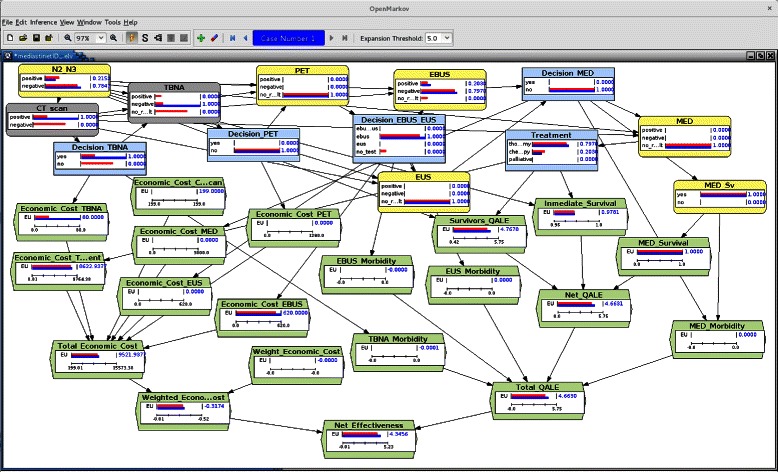
Debugging MEDIASTINET with OpenMarkov. Two evidence cases are displayed in OpenMarkov: the prior case (colored in red), in which no evidence has been introduced, and an evidence case in which the CT scan is positive and the TBNA is negative (colored in blue). Evidential nodes are colored in gray. Bars in each node indicate the posterior probability (chance or decision nodes) or the expected utility (utility nodes) of the corresponding evidence case

All the explanation capabilities for IDs that Elvira and OpenMarkov offer were useful in this quantitative phase of model construction [20, 21]. The display of several evidence cases made it possible to introduce evidence from medical tests and to study its effect on the posterior probability of the variable *N2_N3*. For example, Fig. 3 shows two evidence cases in OpenMarkov: the first one (colored in red) contains no evidence and the probability of each variable is the prior probability; the second case (colored in blue) contains two contradictory findings, as the CT scan is positive and the TBNA is negative. The bars in node *N2_N3* show that the probability of positive *N2_N3* is lower in the second case than the prior probability. When the pneumologist asked us why the model recommended not performing a certain test, we could impose a policy on that test and analyze the outcomes. This phase of debugging was essential for checking the external consistency of the model, as recommended in [31].

## Results

### Computation and representation of optimal strategies

The first step after building the model was to evaluate the reference case of MEDIASTINET, i.e., the version in which every parameter of the model is set to the mean of its associated second-order probability distribution. To have an idea of how big is the search space of evaluating MEDIASTINET, we must note that the size of the set of possible strategies for the set of test decisions is 2.44×10^11^.

Two strategies were computed using two different criteria: maximizing effectiveness (disregarding costs) and maximizing the NHB. The former was carried out by setting *λ* ^−1^ to 0, which according to Eq. 1 means that costs are ignored. In the latter, *λ* ^−1^=1/(30,000 €/QALY) because the shadow willingness-to-pay threshold for the Spanish public health system was estimated to be 30,000 €/QALY [8, 9]. This threshold is consistent with the ones proposed by the World Health Organization for determining whether an intervention is considered cost-effective [32, 33].

Computing these two strategies with different criteria helps to fulfill the recommendation of checking the internal consistency of the model [31] by assigning extreme values for some parameters. In our case, we should expect that the strategy provided by the model when setting *λ* ^−1^ to 0 would tend to select expensive tests that are discarded when *λ*=30,000 €/QALY.

**Fig. 4:**
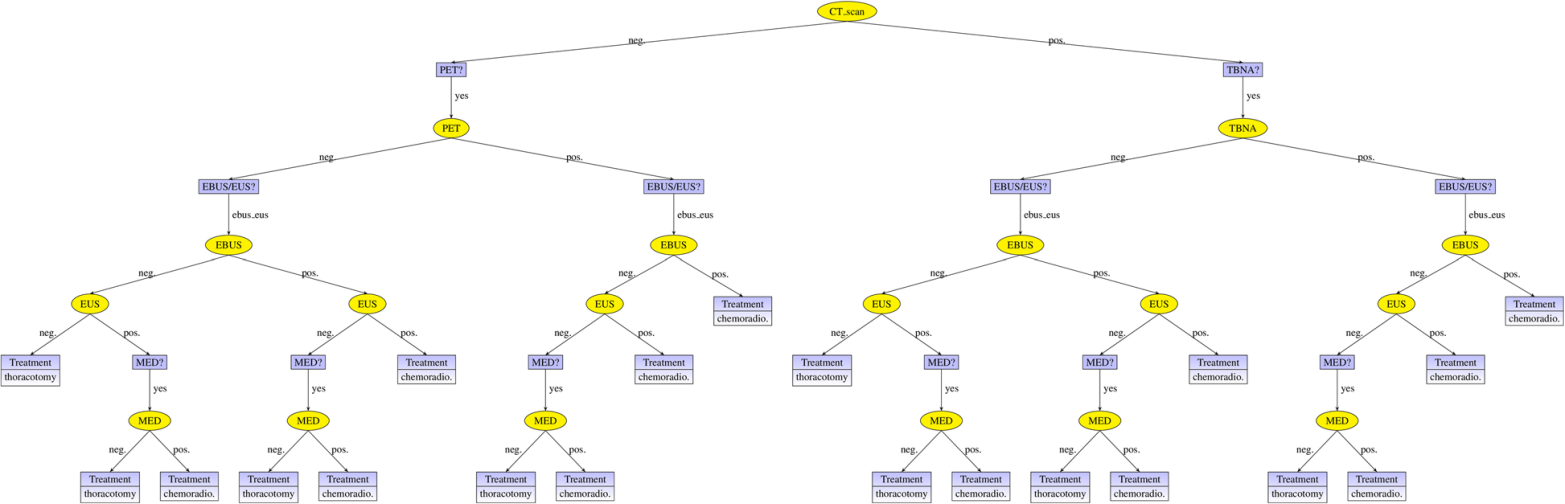
Optimal strategy for MEDIASTINET disregarding costs (*λ* ^−1^=0). When the CT scan is positive, a TBNA, EBUS, and EUS are performed. When the CT scan is negative, a PET, EBUS, and EUS are performed. If the TBNA or the PET is positive, then a mediastinoscopy is performed only if the EBUS and EUS are negative. If the TBNA or the PET is negative, then a mediastinoscopy is performed only if the EBUS and the EUS give contradictory results

**Fig. 5:**
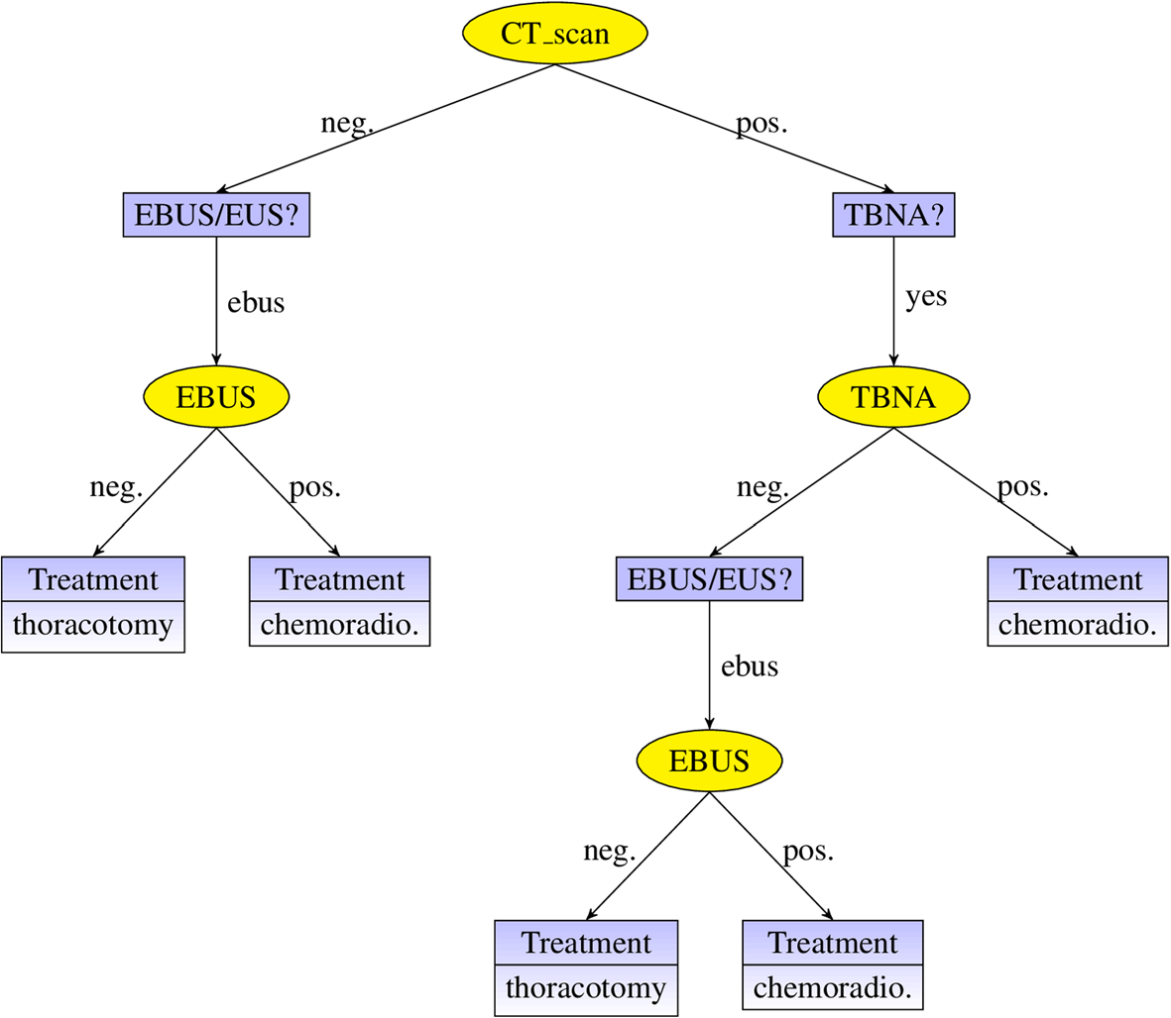
Optimal strategy for MEDIASTINET with costs (*λ* ^−1^=1/(30,000 €/QALY)). A positive CT scan must be followed by a TBNA. An EBUS is done only when the CT scan or the TBNA is negative

These two versions of the ID were evaluated using the variable elimination algorithm [34]. The decision table for each decision grows exponentially with the number of variables known when making the decision. These tables are not an adequate output for a human expert, not only because of their size—for example, the policy table for decision *Treatment* has 15,552 columns—but also because most of the columns correspond to impossible scenarios. For this reason, an algorithm [35] was developed to transform the set of decision tables into a single *strategy tree*, as shown in Figs. 4 and 5. This algorithm builds an auxiliary Bayesian network from the ID by replacing each decision node with a chance node whose probability potential is taken from the corresponding decision table obtained in the evaluation. The strategy tree is then built incrementally by pruning the scenarios incompatible with the policies for previous decisions.

#### Strategy disregarding costs

When costs are disregarded and the CT scan is positive, a TBNA, EBUS, and EUS are performed. If the TBNA is positive and the EBUS and EUS are negative, then doing a mediastinoscopy is recommended; otherwise, a mediastinoscopy is not necessary. If the TBNA is negative, then a mediastinoscopy is performed only if the EBUS and the EUS give contradictory results.

When the CT scan is negative, a PET, EBUS, and EUS are performed. If the PET is positive, then doing a mediastinoscopy is recommended only when the EBUS and EUS are negative. If PET is negative, then a mediastinoscopy is performed only when the EBUS and EUS give contradictory results.

With regards to the treatment, when the TBNA or the PET is positive and EBUS and EUS are the last tests to perform, then the optimal therapy is chemoradiotherapy if the EBUS or EUS is positive, and thoracotomy otherwise. When the TBNA or the PET is negative or when the mediastinoscopy is performed, in all cases the optimal therapy is chemoradiotherapy when the last test is positive and thoracotomy when it is negative.

#### Strategy with costs

When *λ*=30,000 €/QALY, a positive CT scan must be followed by a TBNA. An EBUS is done only when the CT scan or the TBNA are negative. PET is never carried out; put another way, PET is never cost-effective for this value of willingness to pay. The optimal therapy is chemoradiotherapy when the last test is positive and thoracotomy when it is negative.

### Sensitivity analyses

Given the uncertainty of the numerical parameters, three types of sensitivity analyses were performed.

The first type of analysis is based on the *expected value of perfect information* (EVPI); it computes the gain in expected utility that we would obtain if the value of the parameter to study were known with certainty [36]. For the proposed model, the EVPI was lower than 10^−2^ for all parameters except for the specificity of the EBUS when the CT scan is negative.

The second type of analysis is not probabilistic. Let *I* ^∗^ be the optimal strategy of the ID. This analysis consisted of finding the *thresholds of strategy change*, whichdefine the intervals within the parameter variation range such that *I* ^∗^ remains optimal. The thresholds were found by discretizing the parameter variation range and then by determining for each parameter value whether the optimal strategy was identical to *I* ^∗^. This analysis showed that the three parameters with the reference values closest to the strategy change thresholds were, in the following order: the two specificities of the EBUS (when the CT scan is positive and when it is negative) and the specificity of the TBNA when the CT scan is negative. A small variation in the value of the specificity of the EBUS was likely to have an impact because its reference value, 0.995, was very close to the threshold 0.99.

The third type of analysis, *sensitivity of each decision to each parameter*, measured the probability of a change in the optimal strategy given the second-order uncertainty of each parameter. In the proposed model, the three parameters that led to the highest probabilities were, in the following order: (1) the QALE of chemoradiotherapy survivors when there is metastasis, (2) the QALE of thoracotomy survivors when there is metastasis, and (3) the specificity of the EBUS when the CT scan is negative. The decisions most affected by variations in these parameters were, in this order, *Dec_EBUS_EUS* and *Dec_TBNA*.

This third metric is inspired in acceptability curves commonly used in health economic evaluation analyses [27]. An acceptability curve corresponds to a two-axis graph, in which the *X*-axis represents the values in a variation interval of a parameter, and the *Y*-axis represents the probability of a new intervention of being cost-effective in comparison to a control intervention. The third metric presented here computes instead the probability that the optimal intervention remains optimal, i.e. is more cost-effective than any other strategy; then we define the metric as the complementary of such a probability.

The main conclusion of these analyses is that the resulting strategy is robust to the uncertainty of the numerical parameters because only the specificity of the EBUS when the CT scan is negative had a significant impact on the optimal strategy.

### Discussion

When the strategies illustrated in Figs. 4 and 5 were presented to the expert, he agreed with the optimal therapy calculated by the system. He would follow a slightly different strategy that repeats some tests in specific cases (restaging), a possibility that was not considered in this research, but he was not sure whether this strategy is better than that recommended by MEDIASTINET.

Overall, he was favorably impressed by the “intelligence” displayed by this model. In particular, he was surprised that the model recommended TBNA when the CT scan is positive, which differs from current practice, but he thinks that this recommendation should be discussed by the pneumology community because TBNA is a cheap and useful technique that could avoid an EBUS, an expensive test, for many patients. One advantage of using an explicit decision model, publicly available, is that experts can resolve their differences by introducing different parameters into the model and comparing its recommendations. Accordingly, experts from a country other than Spain can introduce different numerical parameters into the model and thereby obtain the results from the perspective of their public health system.

The work presented in this paper has several limitations. First, we have assumed that decisions are totally ordered in MEDIASTINET. However, the optimal strategy could be different if other orderings are allowed in the model. Second, the result of PET after a CT scan can influence the sensitivity and specificity of EBUS, EUS, TBNA and mediastinoscopy; if we removed this assumption of independence (by drawing arcs from PET to such tests), the model could recommend PET in certain scenarios of the strategy with costs. Third, PET can help to detect distant metastasis, which might lead to a different treatment but is beyond the scope of the model. Fourth, some authors are recently combining EBUS and EUS as a single technique where each test analyzes different regions in the mediastinum.

The results were evaluated by a single expert; other experts might disagree with the hypotheses of the model, the numerical parameters, and the assessment of the strategies obtained from the ID. However, the advantage of the recommendations offered in this paper is that they derive from an evidence-based model, which is publicly available, together with an open-source software tool for evaluating it. An expert who disagrees with the conclusions of our study can easily modify the model and analyze the resulting recommendation.

We followed a manual approach for building the ID because the causal relations were very well-known and we did not have enough clinical data; we incorporated all the numerical parameters from the literature. If we have had enough clinical data, other ways of building the model would have been to learn automatically the structure from a data set [37], or to use a hybrid approach, such as *interactive learning* [19], to allow the expert to participate in the model construction. Sesen et al. [38] offer a good study of the application of learning algorithms for the construction of a Bayesian network for lung cancer.

To have an idea of why we chose to build an ID instead of a decision tree, we can give information here about the size of the decision tree. The number of leaves in the symmetric decision tree can be calculated as the product of the number of decision scenarios and the number of ordinary utility nodes. The number of decision scenarios can be calculated as the cardinal of the Cartesian product of the domains of all chance and decision variables [39], which in the case of the proposed ID it is 186,624. The number of ordinary utility nodes in the ID is 15. Thus, the total number of leaves in the symmetric decision tree is 186,624×15=2,799,360, which implies the use of a decision tree is prohibitive in our decision problem.

### Related work

As mentioned in the introduction, Nease and Owens [40] built an ID for the same problem. The model proposed here differs from theirs in several respects. First, MEDIASTINET considers the morbidities of the tests and the cost of tests and therapies, as well as the willingness to pay, *λ*. Four new diagnostic tests that were not routinely used in 1997 [40], have been included in MEDIASTINET, namely *TBNA*, *PET*, *EBUS*,and *EUS*. In line with current practice, MEDIASTINET assumes that a CT scan is always performed. In MEDIASTINET the result of the CT scan influences the sensitivity and specificity of subsequent tests because it gives valuable information about where to stick the needle when doing other tests. One possible treatment is not to treat the pateient, although according to MEDIASTINET it is never the optimal therapy.

As a result of these additions, MEDIASTINET is much bigger and more complex than the model of Nease and Owens [40]. In particular, the decision table for treatment in their model had 72 columns, whereas the number of columns is 15,552 in MEDIASTINET.

Different authors have applied decision trees^3^ to the diagnosis of lung cancer. Different authors [41–44] build different decision trees for assessing the efficacy of PET compared to a baseline strategy that only uses a CT scan. Dietlein et al. [45] performs a similar study, but adding the possibility of performing a mediastinoscopy in certain scenarios. In contrast to these proposals, our model contains additional diagnostic tests (TBNA, EBUS and EUS), and exhaustively studies a much bigger set of strategies.

Closely related to our work, Barosi et al. [46] performed a cost-effectiveness analysis of testing for occult cancer in idiopathic deep vein thrombosis. Their model first decides which cancer to investigate, and, next, for each cancer, it then has two options: (1) to perform a non-invasive test, and, if positive, to perform the most invasive test, and (2) to perform directly the most invasive test. In contrast to our model, Barosi et al. only considered two tests for each cancer, as they had to study a set of different cancers. They only studied two strategies of testing for each cancer, while our model can decide which tests are more appropriate in each scenario, and thus, explore a bigger set of strategies.

Other artificial intelligence approaches have been applied to the diagnosis of lung cancer. Wu et al. [47] selected and grouped some tumor biomarkers through multiple logistic regression, and then used them as inputs for an artificial neural network. Wnuk et al. [48] used an artificial neural network for predicting mediastinal lymph node metastases in NSCLC. Nie et al. [49] studied the diagnosis and prediction of lung cancer of three models with tumor markers based on different classification approaches: logistic regression analysis, decision tree induction and artificial neural networks. Despite the high diagnostic capabilities of these approaches, we think it would still be necessary to integrate them into a framework that can automatically calculate the optimal decisions based on utilities such as costs, life expectancy and willingness-to-pay, similar to what, for example, an ID can do.

## Conclusions

There is a vigorous debate among medical specialists about the optimal sequence of tests for the mediastinal staging of NSCLC [3, 4]. For this reason, we have built MEDIASTINET, a probabilistic model that combines objective data and subjective estimates. It is publicly available and can be evaluated with OpenMarkov. Experts can populate this model with different parameters and analyze whether their differences of opinion are due to discrepancies in the conditional probabilities they are using or to different structural hypotheses. Accordingly, the model proposed here might be a useful decision analysis tool for reaching a consensus.

Moreover, the explicit inclusion of willingness to pay in the proposed model makes it possible to adapt it to each decision-maker; for example, in rich countries *λ* is much higher than in poor regions.

From the perspective of IDs, one contribution of this work is the algorithm for synthesizing huge decision tables into very compact strategy trees, a facility that is not available in other software packages for IDs other than in OpenMarkov.

The quantitative uncertainty in our model has been represented by assigning to each independent parameter the most appropriate distribution, as is usual in the literature. We have applied three metrics for sensitivity analysis: the EVPI of each parameter, the strategy change thresholds, and the sensitivity of each decision to each parameter. These three methods have proven that the proposed model is robust to numerical parameter uncertainty, except the specificity of the EBUS when the CT scan is negative.

As a possibility for future work, we can refine the ID by making the result of PET influence the sensitivity and specificity of subsequent tests. This task would require an in-depth review of the literature due to the exponential growth of the number of parameters required in probability tables.

We may allow partial orderings of the decisions by building a decision analysis network (DAN) [50] instead of an ID. However, the main difficulty would be collecting clinical data to elicit these parameters.

The expert would also like to include in the model the possibility of repeating some diagnostic tests to some patients with N2-positive, previously treated with chemotherapy, to reevaluate them as candidates for surgery, in a process known as *restaging*. For that purpose, various dynamic representations could be considered, such as *partially observable Markov decision processes*[51], and *dynamic limited memory influence diagrams* [52], but again the problem would be the lack of clinical data to estimate the numerical parameters.

Our model assumes that a CT scan is performed on every patient. However, it would be interesting to select a different starting point for the test sequence.

We could constitute a panel of experts to know their opinion about the results of the model. As we mention above, debugging MEDIASTINET with OpenMarkov should help to reach a consensus when experts disagree.

An interesting future work would be to obtain access to a large data set and then to consider learning automatically the model [37], in a similar way to how Sesen et al. [38] built a Bayesian network for lung cancer.

Another line for future research would be to develop new explanation capabilities, which are crucial to facilitate construction of probabilistic decision models and to communicate with experts [20, 21].

## Data availability

The model is publicly available at http://www.probmodelxml.org/networks/id/ID-mediastinet.pgmx. It can be evaluated with OpenMarkov, an open-source software tool that can be downloaded from http://www.openmarkov.org.

## Endnotes

^1^ Ideally, we would also want to minimize other quantitative factors, such as the emotional costs involved for the patient and her/his family, as an ID model for neonatal jaundice did [53]. However, including more criteria apart from monetary and health functions, would require an additional study of the additivity properties in the utility function so that it would still be possible to use the framework of IDs with SVNs.

^2^ Since all costs considered in the model are economic, in the rest of the paper we will just use the term cost to refer to them.

^3^ When mentioning “decision trees” we are referring to the tree-like representation of decision problems under uncertainty [54] used in decision analysis, in contrast to induction decision tree approaches used in automatic learning and classification [55].

## Abbreviations

CI: Confidence interval
CT: Computed tomography
EBUS: Endobronchial ultrasound
EUS: Endoscopic ultrasound
EVPI: Expected value of perfect information
ID: Influence diagram
NHB: Net health benefit
NSCLC: Non-small cell lung cancer
PET: Positron emission tomography
QALE: Quality adjusted life expectancy
QALY: Quality adjusted life year
SVN: Super-value node
TBNA: Transbronchial needle aspiration

## References

[1] Lloyd C, Silvestri GA (2001). Mediastinal Staging of Non-Small-Cell Lung Cancer. Cancer Control.

[2] de Cos Escuín JS, Sorribes LM, Arca JA, Ares AN, Hernández JH, Jover AMC (2006). Estudio multicéntrico epidemiológico-clínico de cáncer de pulmón en España (Estudio Epiclicp-2003). Arch Bronconeumol.

[3] Fritscher-Ravens A, Bohuslavizki KH, Brandt L, Bobrowski C, Lund C, Knofel WT (2003). Mediastinal lymph node involvement in potentially resectable lung cancer. Chest.

[4] Schimmer C, Neukam K, Elert O (2006). Staging of non-small cell lung cancer: clinical value of positron emission tomography and mediastinoscopy. Interact Cardiovasc Thorac Surg.

[5] Sackett DL (1997). Evidence-based medicine. Semin Perinatol.

[6] Friederichs H, Marschall B, Weissenstein A (2014). Practicing evidence based medicine at the bedside: a randomized controlled pilot study in undergraduate medical students assessing the practicality of tablets, smartphones, and computers in clinical life. BMC Med Inform Decis Mak.

[7] Trevena LJ, Zikmund-Fisher BJ, Edwards A, Gaissmaier W, Galesic M, Han PK (2013). Presenting quantitative information about decision outcomes: a risk communication primer for patient decision aid developers. BMC Med Inform Decis Mak.

[8] Sacristán JA, Oliva J, del Llano J, Prieto L, Pinto JL (2002). ¿‘Qué es una tecnología sanitaria eficiente en España?. Gac Sanit.

[9] De Cock E, Miravitlles M, González-Juanatey JR, Azanza-Perea JR (2007). Valor umbral del coste por año de vida ganado para recomendar la adopción de tecnologías sanitarias en España: evidencias procedentes de una revisión de la literatura. Pharmacoeconomics Spanish Res Artic.

[10] Husereau D, Drummond M, Petrou S, Carswell C, Moher D, Greenberg D (2013). Consolidated Health Economic Evaluation Reporting Standards (CHEERS)–explanation and Elaboration: A Report of the ISPOR Health Economic Evaluation Publication Guidelines Good Reporting Practices Task Force. Value Health.

[11] Raiffa H, Schlaifer RO (1961). Applied Statistical Decision Theory.

[12] Howard RA, Matheson JE, Howard RA, Matheson JE (1984). Influence diagrams. Readings on the Principles and Applications of Decision Analysis.

[13] Bielza C, Gómez M, Shenoy PP (2010). Modeling challenges with influence diagrams: Constructing probability and utility models. Decis Support Syst.

[14] Tatman JA, Shachter RD (1990). Dynamic programming and influence diagrams. IEEE Trans Syst Man Cybern.

[15] Nielsen TD, Jensen FV, Laskey K, Prade H (1999). Welldefined decision scenarios. Proceedings of the Fifteenth Conference on Uncertainty in Artificial Intelligence (UAI’99).

[16] Cooper GF (1990). The computational complexity of probabilistic inference using Bayesian belief networks. Artif Intell.

[17] Mauá DD, de Campos CP, Zaffalon M (2012). Solving limited memory influence diagrams. J Artif Intell Res.

[18] Gámez JA, Salmerón A, Elvira Consortium (2002). Elvira: An environment for creating and using probabilistic graphical models. Proceedings of the First European Workshop on Probabilistic Graphical Models (PGM’02).

[19] Bermejo I, Oliva J, Díez FJ, Arias M. Interactive learning of Bayesian networks with OpenMarkov In: Cano A, Gómez M, Nielsen TD, editors. Proceedings of the Sixth European Workshop on Probabilistic Graphical Models (PGM’12). Granada: 2012. p. 27–34.

[20] Lacave C, Oniśko A, Díez FJ (2006). Use of Elvira’s explanation facilities for debugging probabilistic expert systems. Knowl-Based Syst.

[21] Lacave C, Luque M, Díez FJ (2007). Explanation of Bayesian networks and influence diagrams in Elvira. EEE Trans Syst Man Cybern B Cybern.

[22] Drummond MF, Sculpher MJ, Torrance GW, O’Brien BJ, Stoddart GL (2005). Methods for the Economic Evaluation of Health Care Programmes.

[23] Stinnett AA, Mullahy J (1998). Net health benefit: A new framework for the analysis of uncertainty in cost-effectiveness analysis. Med Decis Making.

[24] Silvestri GA, Gonzalez AV, Jantz MA, Margolis ML, Gould MK, Tanoue LT (2013). Methods for staging non-small cell lung cancer: diagnosis and management of lung cancer: American college of chest physicians evidence-based clinical practice guidelines. Chest.

[25] Clarke MG, Kennedy KP, MacDonagh RP (2008). Discussing life expectancy with surgical patients: Do patients want to know and how should this information be delivered?. BMC Med Inform Decis Mak.

[26] Critchfield GC, Willard KE, Connely DP (1986). Probabilistic sensitivity analysis methods for general decision models. Comput Biomed Res.

[27] Briggs A, Claxton K, Sculpher M (2006). Decision Modelling for Health Economic Evaluation.

[28] Gelman A, Carlin JB, Stern HS, Rubin DB (2004). Bayesian Data Analysis.

[29] Bond M, Mealing S, Anderson R, Elston J, Weiner G, Taylor RS (2009). The effectiveness and cost-effectiveness of cochlear implants for severe to profound deafness in children and adults: a systematic review and economic model. Health Technol Assess.

[30] Bond M, Elston J, Mealing S, Anderson R, Weiner G, Taylor R (2010). Systematic reviews of the effectiveness and cost-effectiveness of multi-channel unilateral cochlear implants for adults. Clin Otolaryngol.

[31] Philips Z, Bojke L, Sculpher M, Claxton K, Golder S (2006). Good practice guidelines for decision-analytic modelling in health technology assessment. Pharmacoeconomics.

[32] Edejer TT-T, Baltussen R, Adam T, Hutubessy R, Acharya A, Evans DB (2012). WHO guide to cost-effectiveness analysis. Technical report.

[33] World Health Organization. Choosing interventions that are cost-effective. 2014. http://www.who.int/choice/en/. Accessed 3 Aug 2015.

[34] Luque M, Díez FJ (2010). Variable elimination for influence diagrams with super-value nodes. Int J Approx Reason.

[35] Luque M (2009). Probabilistic graphical models for decision making in medicine. PhD thesis.

[36] Felli JC, Hazen GB (1998). Sensitivity analysis and the expected value of perfect information. Med Dec Making.

[37] Neapolitan RE (2004). Learning Bayesian Networks.

[38] Sesen MB, Nicholson AE, Banares-Alcantara R, Kadir T, Brady M. Bayesian networks for clinical decision support in lung cancer care. PLoS ONE. 2013; 8. doi: 10.1371/journal.pone.0082349. 10.1371/journal.pone.0082349 PMC3855802 24324773

[39] Bielza C, Shenoy PP (1999). A comparison of graphical techniques for asymmetric decision problems. Manag Sci.

[40] Nease RF, Owens KDK (1997). Use of influence diagrams to structure medical decisions. Med Dec Making.

[41] Gambhir S, Hoh C, Phelps M, Madar I, Maddahi J (1996). Decision tree sensitivity analysis for cost-effectiveness of FDG-PET in the staging and management of non-small-cell lung carcinoma. J Nucl Med.

[42] Scott WJ, Shepherd J, Gambhir SS (1998). Cost-effectiveness of FDG-PET for staging non–small cell lung cancer: a decision analysis. Ann Thorac Surg.

[43] Kosuda S, Ichihara K, Watanabe M, Kobayashi H, Kusano S (2000). Decision-tree sensitivity analysis for cost-effectiveness of chest 2-fluoro-2-d-[18f] fluorodeoxyglucose positron emission tomography in patients with pulmonary nodules (non-small cell lung carcinoma) in Japan. Chest.

[44] Wang Y-t, Huang G (2012). Is FDG PET/CT cost-effective for pre-operation staging of potentially operative non-small cell lung cancer?–from Chinese healthcare system perspective. Eur J Radiol.

[45] Dietlein M, Weber K, Gandjour A, Moka D, Theissen P, Lauterbach KW (2000). Cost-effectiveness of FDG-PET for the management of potentially operable non-small cell lung cancer: priority for a PET-based strategy after nodal-negative CT results. Eur J Nucl Med.

[46] Barosi G, Marchetti M, Dazzi L, Quaglini S (1997). Testing for occult cancer in patients with idiopathic deep vein thrombosis–a decision analysis. Thromb Haemost.

[47] Wu Y, Wu Y, Wang J, Yan Z, Qu L, Xiang B (2011). An optimal tumor marker group-coupled artificial neural network for diagnosis of lung cancer. Expert Syst Appl.

[48] Wnuk P, Kowalewski M, Małkowski B, Bella M, Dancewicz M, Szczkesny T, et al. PET-CT derived artificial neural network can predict mediastinal lymph nodes metastases in non-small cell lung cancer patients. Preliminary report and scoring model. Q J Nucl Med Mol Imag. 2014. [Epub ahead of print]. 25289632

[49] Nie GJ, Feng FF, Wu YJ, Wu YM (2009). Diagnosis and prediction of lung cancer through different classification techniques with tumor markers. Chin J Ind Hyg Occup Dis.

[50] Díez FJ, Luque M, König C, Bermejo I (2014). Decision analysis networks. Technical Report CISIAD-14-01.

[51] Åström KJ (1965). Optimal control of Markov decision processes with incomplete state estimation. J Math Anal Appl.

[52] van Gerven MAJ, Díez FJ, Taal BG, Lucas PJF (2007). Selecting treatment strategies with dynamic limited-memory influence diagrams. Artif Intell Med.

[53] Gómez M, Bielza C, Fernández del Pozo JA, Ríos-Insua S (2007). A graphical decision-theoretic model for neonatal jaundice. Med Dec Making.

[54] Raiffa H (1968). Decision Analysis. Introductory Lectures on Choices Under Uncertainty.

[55] Quinlan JR (1986). Induction of decision trees. Mach Learn.

[56] Gould MK, Kuschner WG, Rydzak CE, Maclean CC, Demas AN, Shigemitsu H (2003). Test performance of positron emission tomography and computed tomography for mediastinal staging in patients with non–small-cell lung cancer: a meta-analysis. Annals of Internal Medicine.

[57] Adams K, Shah PL, Edmonds L, Lim E (2009). Test performance of endobronchial ultrasound and transbronchial needle aspiration biopsy for mediastinal staging in patients with lung cancer: systematic review and meta-analysis. Thorax.

[58] Micames CG, McCrory DC, Pavey DA, Jowell PS, Gress FG (2007). Endoscopic ultrasound-guided fine-needle aspiration for non-small cell lung cancer staging: a systematic review and metaanalysis. Chest.

[59] Disdier C. Rentabilidad de la puncion transbronquial en la estadificacion ganglionar del carcinoma pulmonar no microcitico. PhD thesis: Facultad de Medicina. Universidad de Salamanca; 2001.

[60] Herth FJ, Eberhardt R, Krasnik M, Ernst A (2008). Endobronchial ultrasound-guided transbronchial needle aspiration of lymph nodes in the radiologically and positron emission tomography-normal mediastinum in patients with lung cancer. Chest.

[61] Szlubowski A, Zieliński M, Soja J, Annema JT, Sośnicki W, Jakubiak M (2010). A combined approach of endobronchial and endoscopic ultrasound-guided needle aspiration in the radiologically normal mediastinum in non-small-cell lung cancer staging—a prospective trial. Eur J Cardiothorac Surg.

[62] ORDEN 731/2013, de 6 de septiembre, del Consejero de Sanidad, por la que se fijan los precios públicos por la prestación de los servicios y actividades de naturaleza sanitaria de la Red de Centros de la Comunidad de Madrid. Boletín Oficial de la Comunidad de Madrid, no. 215. 2013. https://www.bocm.es/boletin/CM_Orden_BOCM/2013/09/10/BOCM-20130910-1.PDF.

[63] Gómez León N, Escalona S, Bandrés B, Belda C, Callejo D, Blasco JA (2014). ^18^f-fluorodeoxyglucose positron emission tomography/computed tomography accuracy in the staging of non-small cell lung cancer: Review and cost-effectiveness. Radiol Res Pract.

[64] Holty JEC, Kuschner WG, Gould MK (2005). Accuracy of transbronchial needle aspiration for mediastinal staging of non-small cell lung cancer: a meta-analysis. Thorax.

[65] Castelao Naval J, Alonso JLI, Carrasco JG, Hernández IS, Sánchez CA, Francés JF (2013). Clinical utility and economic impact of conventional transbronchial needle aspiration of mediastinal lymphadenopathies in bronchogenic carcinoma. Arch Bronconeumol (English Edition).

[66] Von Bartheld MB, Van Breda A, Annema JT (2014). Complication rate of endosonography (endobronchial and endoscopic ultrasound): a systematic review. Respiration.

[67] Kunst PWA, Eberhardt R, Herth FJF (2008). Combined ebus real time tbna and conventional tbna are the most cost-effective means of lymph node staging. J Bronchology Interv Pulmono.

[68] Navani N, Spiro SG, Janes SM (2009). Mediastinal staging of NSCLC with endoscopic and endobronchial ultrasound. Nat Rev Clin Oncol.

[69] Silvestri GA, Gould MK, Margolis ML, Tanoue LT, McCrory D, Toloza E (2007). Noninvasive staging of non-small cell lung cancer: Accp evidenced-based clinical practice guidelines. Chest.

[70] Ramnath N, Dilling TJ, Harris LJ, Kim AW, Michaud GC, Balekian AA (2013). Treatment of stage iii non-small cell lung cancer. diagnosis and management of lung cancer, 3rd ed: American college of chest physicians. evidence-based clinical practice guidelines. Chest.

[71] Meyers BF, Haddad F, Siegel BA, Zoole JB, Battafarano RJ, Veeramachaneni N (2006). Cost-effectiveness of routine mediastinoscopy in computed tomography–and positron emission tomography–screened patients with stage i lung cancer. J Thorac Cardiovasc Surg.

[72] Malenka DJ, Colice G, Beck JR (1991). Does the mediastinum of patients with non-small cell lung cancer require histologic staging?. Am Rev Respir Dis.

[73] Detterbeck FC, Gibson CJ (2008). Turning gray: The natural history of lung cancer over time. J Thorac Oncol.

[74] European Society of Thoracic Surgeons. Database Annual Report. 2015. http://www.ests.org/collaboration/database_reports.aspx. Accessed 3 Aug 2015.

[75] Corral J, Espinàs JA, Cots F, Pareja L, Solà J, Font R (2015). Estimation of lung cancer diagnosis and treatment costs based on a patient-level analysis in Catalonia (Spain). BMC Health Serv Res.

